# Statistical Variability and Lower-Tail Performance Assessment of Tensile Properties in Flax, Jute, and Carbon Fiber Composite Laminates

**DOI:** 10.3390/polym18141746

**Published:** 2026-07-16

**Authors:** Saurabh Tiwari, Jongwon Lee, Mohammad Faseeulla Khan, Nokeun Park

**Affiliations:** 1 School of Materials Science and Engineering, Yeungnam University, Gyeongsan 38541, Republic of Korea; 2 Department of Mechanical Engineering, College of Engineering, King Faisal University, Al-Ahsa 31982, Saudi Arabia; 3 Institute of Materials Technology, Yeungnam University, Gyeongsan 38541, Republic of Korea

**Keywords:** natural fiber composites, flax, jute, tensile properties, coefficient of variation, lower-tail reliability, weibull distribution

## Abstract

Natural fiber-reinforced polymer composites are attractive for lightweight and sustainable engineering applications; however, property scatter remains a major barrier to reliable design. Mean tensile properties alone are insufficient when material selection depends on repeatability and lower-tail performance. This study presents a statistical variability and lower-tail reliability assessment of flax, jute, and carbon fiber composite laminates using 590 open-access tensile test records from a published natural-fiber composite dataset. Flax and jute were selected as representative bast-fiber systems covering a range of woven, unidirectional, and short-fiber architectures; carbon fiber was included as a synthetic-fiber reference system. Three mechanically important properties were analyzed: the recalculated tensile modulus, tensile strength, and axial failure strain. Normal, lognormal, and two-parameter Weibull distributions were screened for each material–property combination using the Akaike information criterion (AIC); empirical fifth percentiles (P5) and bootstrap 95% confidence intervals (CI) were computed as lower-tail descriptors. The results show that Carbon-0 has the highest lower-tail modulus and strength, with empirical fifth percentiles of 104.95 GPa and 989.64 MPa, respectively. Among the natural fiber systems, Flax-0 and Flax-VE-0 provided the highest lower-tail strengths, whereas Flax-Twill and Flax-CP showed the highest lower-tail failure strains. The lowest tensile strength coefficient of variation was observed for Flax-90 (2.41%), followed by Flax-Twill (3.43%), Flax-0 (4.50%), Jute-Satin (4.83%), and Jute-Plain (4.92%). A balanced reliability ranking that combined lower-tail property ranks and coefficient of variation ranks identified Flax-0, Flax-VE-0, Flax-Twill, Flax-CP, and Jute-Satin as the most favorable natural-fiber systems. The lower coefficient of variation values observed in aligned and satin-weave architectures relative to short-fiber and plain-weave systems reflect the role of fiber orientation uniformity in moderating property scatter at the laminate scale. This study provides a reproducible statistical framework based on lower-tail performance descriptors for comparative screening purposes, not on formal design allowables for distinguishing high mean performance from reliable minimum-level performance in natural fiber composite laminates.

## 1. Introduction

Natural fiber-reinforced polymer composites are increasingly being studied as lower-density and more sustainable alternatives to conventional synthetic-fiber composites. Flax and jute fibers are particularly attractive because they are renewable, low-density, and capable of providing useful stiffness and strength when aligned or woven in polymer matrices. Recent reviews have emphasized that these materials are promising for structural and semi-structural applications; however, their broader adoption is limited by moisture sensitivity, architecture dependence, manufacturing variability, and scatter in mechanical properties [1,2,3]. While carbon and glass fiber reinforced polymer composites offer superior absolute tensile strength and stiffness, natural-fiber composites provide competitive specific properties on a strength-to-density basis, significantly lower raw material and processing costs, biodegradability at end of life, and a substantially reduced carbon footprint during production [4,5]. The production cost of natural-fiber composite components is typically 25–60% lower than equivalent glass-fiber systems, making them attractive for cost-sensitive structural and semi-structural applications in the automotive, construction, and packaging sectors [5]. However, natural fibers are inherently more variable than synthetic fibers owing to biological origin, and this variability is amplified by differences in extraction route, textile architecture, matrix compatibility, and moulding process parameters including cure temperature, consolidation pressure, and fiber volume fraction [6]. These factors collectively govern both the mean property level and the scatter of the resulting laminate properties, and their combined influence motivates the statistical treatment presented in this study. Among the studied architectures, woven fabrics, particularly satin and twill weaves, have attracted interest because their interlaced fiber structure can improve damage tolerance relative to unidirectional laminates, although at the cost of reduced in-plane stiffness and strength [7]. Short-fiber systems, while easier to manufacture, typically exhibit greater property scatter owing to random fiber orientation and the sensitivity of stress transfer to the local fiber length distribution [8].

In engineering design, the average tensile modulus or strength is only one part of the material-selection problem. A material with a high mean strength but high scatter may be less attractive than a material with a lower mean but more stable performance. This issue is particularly important for natural fiber systems because biological fibers vary with the plant source, extraction route, fiber morphology, textile architecture, and composite processing conditions [6]. The variability of flax fibers and flax-based reinforcements has been discussed as a central challenge for composite reinforcement, even when a controlled fiber supply and optimized processing can reduce scatter [6]. Weibull statistics have been applied to characterize the strength variability of individual flax and jute fibers, and the resulting shape parameters provide a measure of the concentration or dispersion of the strength population [9]. However, the transition from single-fiber variability to composite laminate variability is not straightforward, as fiber averaging effects, matrix constraints, and textile architecture collectively moderate the scatter observed at the laminate scale [10].

Composite design practices also recognize the importance of lower-tail properties. In aerospace and structural composite qualification, design allowables are commonly derived from statistical descriptions of coupon data rather than from the mean alone [11,12]. The present dataset is not a formal qualification dataset, and the values reported here are not certified A-basis or B-basis allowable. However, lower-tail descriptors, such as empirical fifth percentiles, are still useful for comparing material systems and identifying candidates that combine high performance with low scatter. The coefficient of variation has been used as a practical repeatability index in natural fiber composite studies, with values below 10% generally considered acceptable for structural screening purposes and values above 15% indicating material systems that require process improvement before design use [13].

Torres et al. published a large open-access tensile dataset comprising flax, jute, and carbon fiber composite laminates with unidirectional, cross-ply, short-fiber, and woven architectures [14]. Their related statistical investigation showed that long natural-fiber composites can exhibit variability levels comparable to carbon-fiber systems under controlled testing conditions [10]. The breadth and quality of this dataset make it particularly suitable for a reliability-based reanalysis focused on statistical variability, lower-tail performance, and the comparative reliability of different composite laminate systems. The objective of this study was to quantify and compare the statistical variability and lower-tail reliability of tensile modulus, tensile strength, and axial failure strain across ten composite laminate systems. Using a dataset comprising 590 individual tensile-test records, the study provides a reproducible group-wise assessment of material variability through statistical descriptors and coefficient-of-variation analyses. Lower-tail performance was evaluated using both empirical and distribution-based fifth percentile estimates, while normal, lognormal, and two-parameter Weibull distributions were examined to identify the most appropriate reliability models for each material–property combination. Based on these analyses, a balanced reliability ranking was developed to support material selection where both mechanical performance and consistency are important. Unlike the original statistical study by Torres et al., which reported group-level means and standard deviations and identified fiber type and architecture as the primary sources of property variability [10], the present work extends the dataset in three important ways. First, it quantifies lower-tail performance using empirical fifth percentiles and bootstrap confidence intervals, which were not included in the original analysis. Second, it performs systematic AIC-based screening of Normal, lognormal, and Weibull distributions across all three tensile properties and ten material groups, rather than assuming a universal distribution. Third, it integrates multi-property lower-tail behavior into a balanced reliability ranking with sensitivity analysis to weighting choice. Unlike prediction-oriented machine-learning studies, this work focuses on a reproducible statistical framework for lower-tail reliability assessment and comparative material screening using an open natural-fiber composite dataset.

## 2. Materials and Dataset

### 2.1. Data Source

The raw data were obtained from the open dataset associated with the article “Statistical data for the tensile properties of natural fiber composites” [14] and the corresponding Mendeley Data record [14]. This dataset supports the research article “The mechanical properties of natural fiber composite laminates: A statistical study” [10]. The previous reported work [10,14] all relate to the same experimental program; they correspond to the companion research article, the data article, and the Mendeley Data repository, respectively, for a single cohesive dataset comprising 590 tensile tests conducted under consistent laboratory conditions. The restriction to flax and jute composites reflects the scope of this well-characterized open-access experimental dataset, which was selected for its size, data quality, and experimental consistency. The statistical framework developed in this study is readily applicable to other natural-fiber composite systems when comparable datasets become available. The raw files contain specimen metadata and tensile test time-series data, including time, extension, load, video axial strain, video transverse strain, and calculated stress.

### 2.2. Material Groups

**Figure A1:**
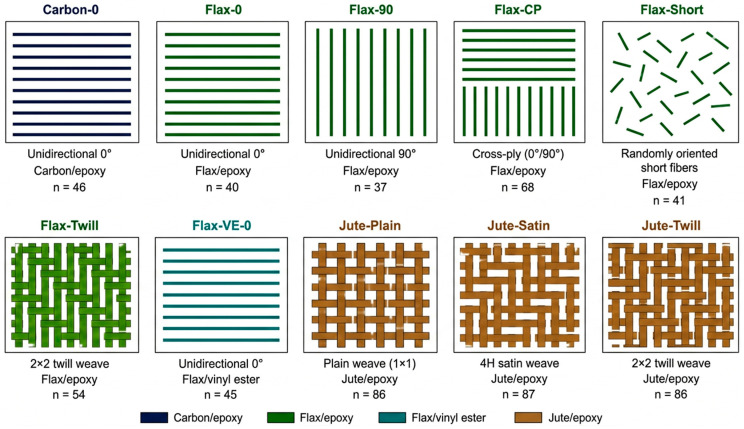
Schematic representations of the laminate architectures for the ten composite material groups included in the dataset. The schematics illustrate fiber orientation and weave architecture only and are not drawn to scale. All material groups employed an epoxy thermoset matrix except Flax-VE-0, which used a vinyl ester matrix. Carbon-0 is included as the synthetic-fiber reference system. Specimen counts (n) are shown for each material group.

The analysis included 590 valid specimens across ten material groups: Carbon-0, Flax-0, Flax-90, Flax-CP, Flax-Short, Flax-Twill, Flax-VE-0, Jute-Plain, Jute-Satin, and Jute-Twill. These groups represent the carbon, flax, and jute fiber systems with unidirectional, transverse, cross-ply, short-fiber, and woven architectures. The number of specimens per group ranged from 37 to 87. All material groups except Flax-VE-0 employed an epoxy thermoset matrix, whereas Flax-VE-0 was manufactured using a vinyl ester matrix to enable comparison of matrix chemistry effects within the same unidirectional (0°) flax–fiber architecture. Both epoxy and vinyl ester are widely used thermoset resins in structural composite applications. Throughout this study, the designation Flax-0 refers to the epoxy-matrix unidirectional flax laminate (equivalent to Flax-EP-0), while Flax-VE-0 is retained to distinguish the vinyl ester system. Carbon-0 denotes a unidirectional (0°) carbon fiber/epoxy laminate and is the only carbon-fiber group included in the source dataset. It is incorporated as a synthetic-fiber reference to provide performance and variability benchmarks for comparison with the natural-fiber composite systems. Schematic illustrations of the ten laminate architectures are provided in Appendix A Figure A1. A file-level audit confirmed that all 590 raw CSV files were represented exactly once in the processed specimen table with no missing or duplicate entries.

### 2.3. Retained Properties

Three tensile properties were retained for analysis: tensile modulus, tensile strength, and axial failure strain. The tensile modulus was recalculated from the raw stress–strain curves using the strain intervals defined by Torres et al. [10,14]. Tensile strength was determined as the stress corresponding to the peak load, while axial failure strain was obtained from the video-based axial strain measurement at peak load, with extension-derived strain used only when video strain data were unavailable. Poisson’s ratio and toughness were excluded from the present analysis. For context, the tensile properties reported in this study represent composite laminate properties rather than individual fiber properties. Elementary flax fibers have reported tensile strengths of approximately 1.5–1.8 GPa and Young’s moduli of 50–80 GPa, whereas elementary jute fibers typically exhibit tensile strengths of 200–800 MPa and moduli of 20–55 GPa [8]. The substantially lower laminate strengths observed in the present study are expected and are consistent with composite mechanics, reflecting the combined effects of fiber volume fraction, fiber orientation efficiency, fiber–matrix stress transfer, and processing-related imperfections such as voids and fiber misalignment. The focus of this study is on the primary tensile properties that are most relevant to structural performance, reliability assessment, and material selection, and that can be consistently extracted across the entire dataset.

## 3. Methods

### 3.1. Data Processing and Statistical Analysis

Each CSV file was processed using a reproducible workflow implemented in Python 3.11.x (Python Software Foundation; https://www.python.org, accessed 2 June 2026). Specimen geometry, material identifiers, and laminate descriptors were extracted from the metadata and folder structure, while the tensile response was obtained from the corresponding time-series data. The workflow generated a single specimen-level record for each tensile test, providing the basis for subsequent statistical analyses. For each material group and retained tensile property, descriptive and variability statistics were calculated, including the number of specimens (*n*), mean, standard deviation, coefficient of variation (CV), median, interquartile range, 95% confidence interval of the mean, empirical fifth percentile, and bootstrap 95% confidence interval of the empirical fifth percentile. The coefficient of variation was calculated as the ratio of the standard deviation to the mean and expressed as a percentage. This metric was used as a normalized measure of relative scatter and within-group consistency. The coefficient of variation (CV) is an indicator of relative dispersion and property stability rather than a formal engineering reliability metric, which requires knowledge of loading conditions and target failure probabilities. The empirical fifth percentile (P_5_) was selected as the primary lower-tail descriptor because it represents the value below which only 5% of observations fall, thereby providing a conservative estimate of minimum performance consistent with the philosophy of B-basis design allowables for composite structures [11,12]. Bootstrap confidence intervals for P_5_ were estimated using 10,000 resamples to quantify the uncertainty associated with finite sample sizes (n = 37–87). In addition to conventional statistical descriptors (mean, standard deviation, and CV), the present framework incorporates (i) bootstrap confidence intervals for lower-tail estimates, (ii) AIC-based screening of normal, lognormal, and Weibull distributions for each material–property group, and (iii) a balanced multi-property ranking with sensitivity analysis. Together, these analyses provide a reproducible framework for lower-tail reliability assessment and comparative screening of natural-fiber composite laminates.

### 3.2. Distribution Screening

Normal, lognormal, and two-parameter Weibull distributions were fitted to each material–property group. Normal and lognormal distributions were estimated from their standard closed-form parameters. The Weibull distribution was estimated using a linear rank regression probability plot approach. The normal and lognormal distributions were fitted using maximum likelihood estimation (MLE), while the two-parameter Weibull distribution was fitted using linear rank regression on Weibull probability plots with Benard’s median-rank plotting positions,

*F_i_* = (*i* − 0.3)/(*n* + 0.4) (1)

where *i* is the specimen rank and *n* is the sample size. Distribution selection was performed using the Akaike information criterion (AIC),

(2) $$\mathrm{AIC}=-2\ln(\hat{L})+2k,$$

where $\hat{L}$ is the maximized likelihood and *k* is the number of model parameters (two for all distributions considered), ensuring a fair comparison. Lower AIC values indicate better empirical support. Following established guidelines, distributions with AIC differences (ΔAIC) smaller than 2 were considered to have comparable support [15]. In such cases, the empirical fifth percentile was retained as the principal lower-tail descriptor. Since different fiber architectures and manufacturing routes can produce distinct statistical populations, each material–property group was evaluated independently rather than assuming a universal distribution model. When two distributions had AIC values within two units of each other, both were considered adequately supported, and the empirical fifth percentile was used as the primary lower-tail descriptor, regardless of the fitted model. The distribution-based fifth percentile was calculated from the best AIC-ranked distributions. These values were reported as screening-level lower-tail descriptors. They are not certified design allowable because formal allowable generation requires additional assumptions, batch structures, outlier handling, and qualification procedures [11,12].

### 3.3. Balanced Reliability Ranking and Sensitivity Analysis

A balanced reliability ranking was developed to compare the material systems across the three retained tensile properties. For each property, the material groups were ranked according to their empirical fifth percentile values, with higher lower-tail performance receiving a better rank. The groups were also ranked according to their coefficients of variation (CV), where lower CV values received a better rank. The overall reliability score was then calculated as: composite rank score = 0.65 × mean lower-tail rank + 0.35 × mean CV rank.

Lower composite scores indicate a more favorable combination of lower-tail performance and repeatability. The weighting scheme was selected to reflect the primary objective of the study, namely the identification of material systems that maintain reliable minimum-level properties while exhibiting acceptable variability. Greater emphasis was therefore placed on lower-tail performance (65%), whereas repeatability represented by the CV contributed 35% of the final score. This approach prioritizes materials that deliver dependable minimum performance while penalizing systems exhibiting excessive scatter. To evaluate the robustness of the ranking procedure, a sensitivity analysis was performed using three weighting combinations: 50/50, 65/35, and 80/20 between lower-tail performance and CV ranking. Consistent rankings across these scenarios were interpreted as evidence that the conclusions were not strongly dependent on a particular weighting choice. The proposed ranking should be viewed as a transparent comparative screening tool for the present dataset rather than a universal design index. Readers should note that the composite ranking is primarily intended for within-class comparison of the natural-fiber composite systems. Carbon-0 is included as a synthetic-fiber reference to provide performance context and to demonstrate the ability of the ranking framework to distinguish different lower-tail performance profiles. Owing to the fundamentally different mechanical characteristics of carbon- and natural-fiber composites, direct comparison should be interpreted with appropriate caution.

## 4. Results

### 4.1. Coefficient of Variation Across Material Groups

**Figure 1:**
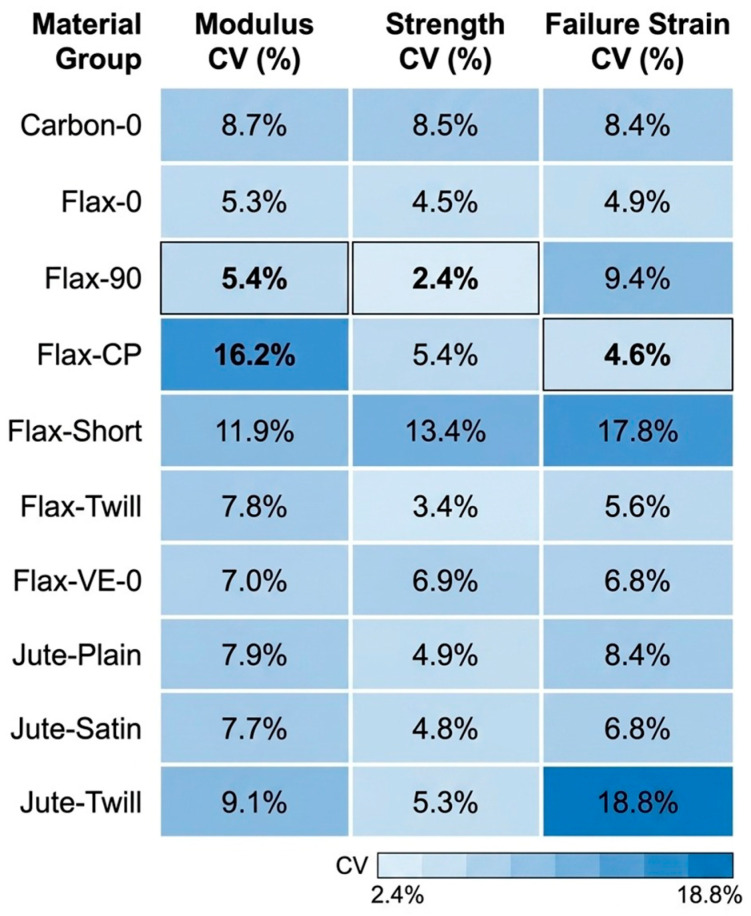
Coefficient of variation (CV) for recalculated tensile modulus, tensile strength, and axial failure strain across the investigated composite laminate systems. Lower CV values indicate greater repeatability and reduced relative scatter within a material group.

Figure 1 summarizes the coefficients of variation (CVs) for the three retained tensile properties across all material groups. Overall, the CV values ranged from 2.41% to 18.80%, indicating substantial differences in repeatability among the composite laminate systems investigated. Tensile strength exhibited relatively low variability in several groups. Flax-90 showed the lowest tensile-strength CV (2.41%), followed by Flax-Twill (3.43%), Flax-0 (4.50%), Jute-Satin (4.83%), and Jute-Plain (4.92%). In contrast, Flax-Short exhibited the highest tensile-strength CV (13.42%), indicating considerable scatter in strength response. For tensile modulus, Flax-CP showed the highest variability (16.16%), followed by Flax-Short (11.87%) and Jute-Twill (9.11%). Failure strain displayed the greatest variability overall, with Jute-Twill (18.80%) and Flax-Short (17.76%) exhibiting the highest CV values, whereas Flax-CP (4.58%) and Flax-0 (4.87%) exhibited the lowest relative scatter. Carbon-0 showed consistently moderate CV values across all three properties (8.7% modulus, 8.5% strength, and 8.4% failure strain), indicating relatively stable tensile behavior within the tested dataset. Notably, five natural-fiber laminate systems—Flax-90, Flax-Twill, Flax-0, Jute-Satin, and Jute-Plain—exhibited tensile-strength CV values below 5%, demonstrating that well-designed natural-fiber composites can achieve high levels of repeatability despite the inherent variability commonly associated with biological reinforcement materials.

The observed differences in CV among the material groups reflect the strong influence of fiber architecture and manufacturing on composite variability. Randomly oriented short-fiber composites (Flax-Short) exhibited the highest scatter because variations in fiber length, orientation, and local resin-rich regions result in heterogeneous stress transfer and failure initiation. Cross-ply laminates (Flax-CP) showed relatively high modulus variability owing to the sensitivity of laminate stiffness to ply orientation, ply thickness, and consolidation quality. In contrast, unidirectional laminates (Flax-0 and Flax-90) and well-controlled woven architecture (e.g., Flax-Twill and Jute-Satin) exhibited lower scatter because of more uniform fiber alignment and load transfer. Carbon-0 displayed consistently moderate CV values across all three tensile properties, reflecting the greater manufacturing consistency and tighter quality control of industrial carbon fibers compared with natural-fiber reinforcements.

### 4.2. Tensile Strength Distributions

**Figure 2:**
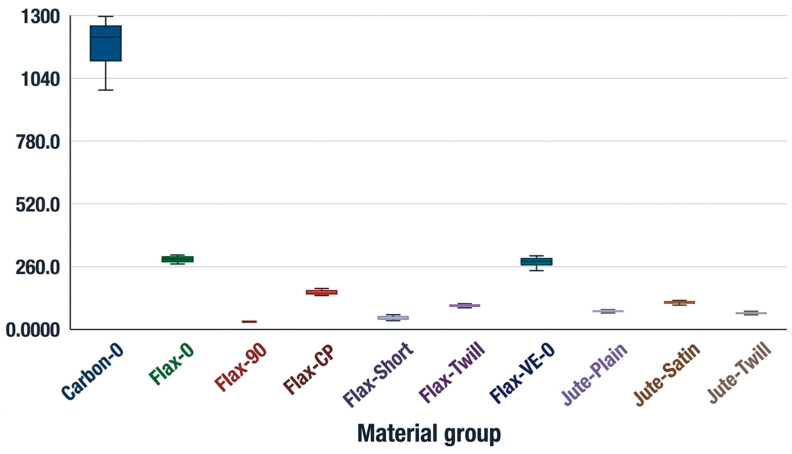
Tensile strength distributions according to the material group. Boxes indicate the interquartile range, the internal line indicates the median, and whiskers indicate the 5th–95th percentile range.

Figure 2 shows the tensile strength distribution. Carbon-0 had the highest strength by a large margin, with a mean strength of 1176.11 MPa and an empirical fifth percentile of 989.64 MPa. Among the natural fiber systems, Flax-0 and Flax-VE-0 had the highest tensile strength, with empirical fifth percentiles of 268.94 MPa and 241.26 MPa, respectively. Flax-CP was followed by a lower-tail strength of 141.88 MPa. The woven jute groups exhibited moderate strength but relatively consistent distributions. The observed laminate tensile strengths are substantially lower than the intrinsic strengths of individual flax and jute fibers because composite performance is governed by fiber volume fraction, fiber orientation efficiency, fiber–matrix stress transfer, and processing-related imperfections at the laminate scale. The marked differences among the flax systems primarily reflect fiber architecture. The unidirectional Flax-0 laminate exhibited the highest strength because the fibers are aligned with the loading direction, enabling efficient load transfer. In contrast, the lower strengths of the woven (Flax-Twill) and randomly oriented short-fiber (Flax-Short) laminates arise from fiber crimp, orientation effects, and less efficient stress transfer, which reduce the effective load-carrying capacity. Similarly, the higher tensile strength of Jute-Satin compared with Jute-Plain is attributed to the lower fiber crimp and longer float lengths of the satin weave, allowing a greater proportion of fibers to align with the loading direction. The observed group strengths are consistent with the ranges reported for comparable natural-fiber composite laminates in the literature [1,8]. Jute-Satin had the highest jute strength, with a mean of 107.87 MPa and an empirical fifth percentile of 98.94 MPa. The ratio of empirical fifth percentile to mean strength, an indicator of how far the lower tail departs from the central tendency, ranged from 0.79 for Flax-VE-0 to 0.95 for Jute-Satin, with most natural-fiber groups falling between 0.88 and 0.93. This narrow range suggests that, despite differences in absolute strength, the relative shape of the lower-tail distribution is broadly similar across the woven and cross-ply natural fiber systems in this dataset.

### 4.3. Strength-Modulus-Failure Strain Trade-Off

**Figure 3:**
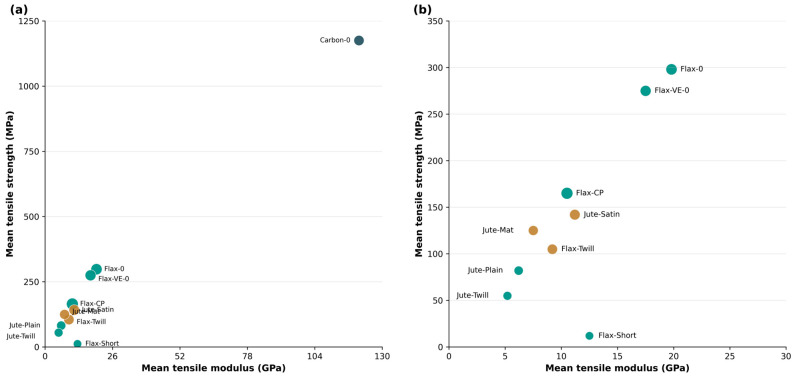
Relationship between mean tensile modulus and mean tensile strength. Bubble size is proportional to the empirical fifth percentile (P_5_) of axial failure strain. (**a**) All ten material groups, including Carbon-0 as a synthetic-fiber reference. (**b**) Enlarged view of the natural-fiber composite region to improve visual comparison among the flax and jute systems.

Figure 3a,b illustrate the relationship between mean tensile modulus and mean tensile strength for the investigated composite systems. Carbon-0 is included in Figure 3a as a synthetic-fiber reference to (i) contextualize the performance of natural-fiber composites against an industrial benchmark, (ii) illustrate the characteristic trade-off between stiffness/strength and failure strain, and (iii) provide a reference for comparing the variability of mature carbon-fiber composites with that of natural-fiber systems. Since Carbon-0 occupies a distinct high-stiffness, high-strength region, it compresses the visual separation among the natural-fiber groups. Therefore, Figure 3b presents an enlarged view of the natural-fiber region to facilitate comparison of the flax and jute composite systems. Carbon-0 occupies a distinct region characterized by substantially higher stiffness and strength than all natural-fiber laminate groups. Among the natural-fiber systems, Flax-0 and Flax-VE-0 exhibit the highest combinations of modulus and strength, whereas Flax-CP and Flax-Twill achieve lower strength levels but exhibit comparatively greater lower-tail failure strain. These results highlight the trade-off between stiffness, strength, and deformation capability within the dataset. Consequently, material selection should consider the specific performance requirements of the intended application, as systems optimized for maximum stiffness and strength do not necessarily provide the highest strain tolerance. The results further demonstrate that lower-tail reliability assessment benefits from evaluating multiple tensile properties rather than relying on a single performance metric.

### 4.4. Lower-Tail Modulus, Strength, and Failure Strain

**Figure 4:**
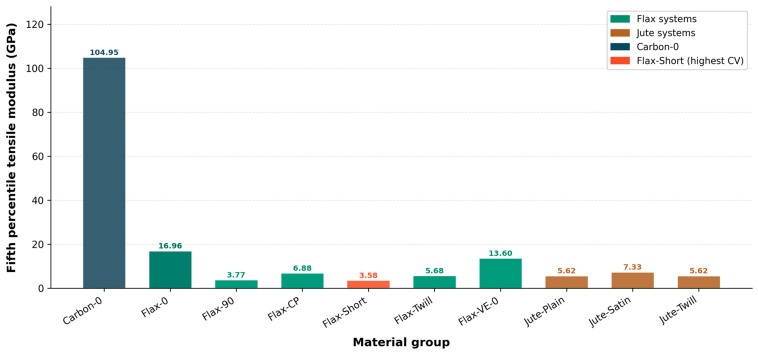
Empirical fifth percentile of the recalculated tensile modulus by material group.

**Figure 5:**
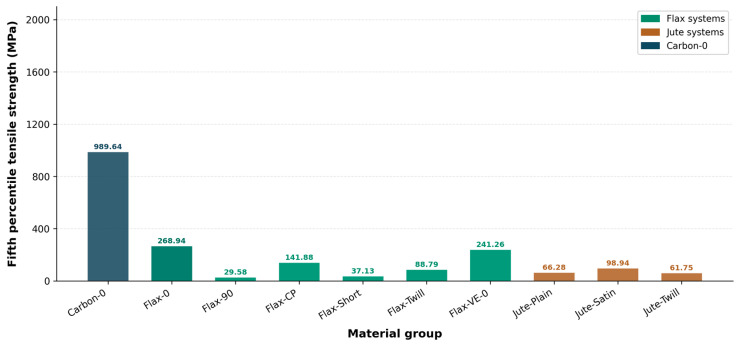
Empirical fifth percentile of tensile strength for each material group.

**Figure 6:**
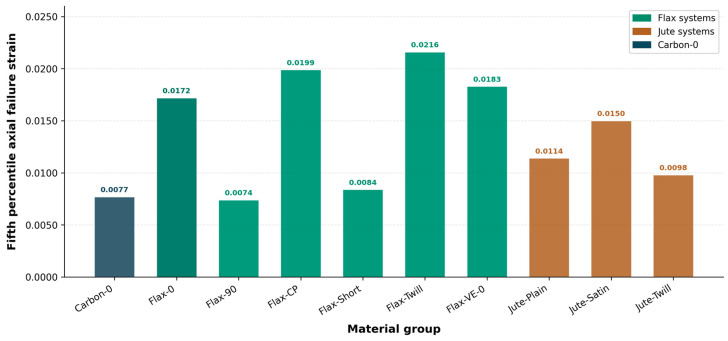
Empirical fifth percentile of axial failure strain for each material group. Higher values indicate greater lower-tail deformation capability.

Figure 4, Figure 5 and Figure 6 show the empirical fifth percentile values for the three target properties. Carbon-0 exhibited the highest lower-tail modulus and strength. Among the natural fiber systems, Flax-0 exhibited the highest lower-tail modulus and strength, followed by Flax-VE-0. Jute-Satin was the strongest jute system in the lower tail region. The failure strain exhibited a different ranking.

Flax-Twill had the highest empirical fifth percentile of failure strain (0.0216), followed by Flax-CP (0.0199), Flax-VE-0 (0.0183), Flax-0 (0.0172), and Jute-Satin (0.0150). Carbon-0 had high stiffness and strength but a lower failure strain fifth percentile (0.0077), consistent with a stiffer and more brittle tensile response. Notably, Flax-CP, despite having the highest modulus CV (16.16%) among all groups, achieved a competitive lower-tail failure strain of 0.0199, which is the second highest among all ten systems. This indicates that a high modulus scatter does not necessarily translate into a high failure strain scatter, and that these two properties carry independent information for reliability assessment. Designers prioritizing damage tolerance should consider the failure strain lower-tail performance separately from the stiffness repeatability.

### 4.5. Distribution Screening and Weibull Reliability Behavior

Table 1 summarizes the empirical and best-fit model fifth percentile values for the tensile strength. The present study evaluates all three retained tensile properties tensile modulus, tensile strength, and axial failure strain. Table 1 presents the distribution screening and lower-tail results for tensile strength, while the corresponding analyses for tensile modulus and axial failure strain are summarized in Table 2. All three properties are subsequently integrated into the balanced reliability ranking described in Section 3.3 and Section 4.6. The use of different best-fit distributions across material groups does not compromise the comparability of the lower-tail estimates. Instead, AIC-based model selection allows each material–property group to be represented by the distribution that best describes its statistical behavior. Regardless of the selected distribution (normal, lognormal, or Weibull), the estimated fifth percentile represents the same lower-tail performance metric and is therefore directly comparable across all material groups. For tensile strength, the normal distribution was selected by the AIC for Flax-0, Flax-CP, Flax-Twill, Jute-Plain, and Jute-Satin. The Weibull distribution was selected for Carbon-0, Flax-90, Flax-VE-0, and Jute-Twill, whereas the lognormal distribution was selected for Flax-Short. The model-based fifth percentiles were generally close to empirical values, supporting their use as screening-level, lower-tail descriptors.

Table 2 lists the corresponding best-fit distribution classes and empirical fifth percentile values for the modulus and failure strain. The modulus fits were predominantly lognormal, whereas the failure strain fits varied among normal, lognormal, and Weibull forms. This reinforces the need to screen distributions separately for each property rather than applying the tensile strength fit pattern to all targets.

**Figure 7:**
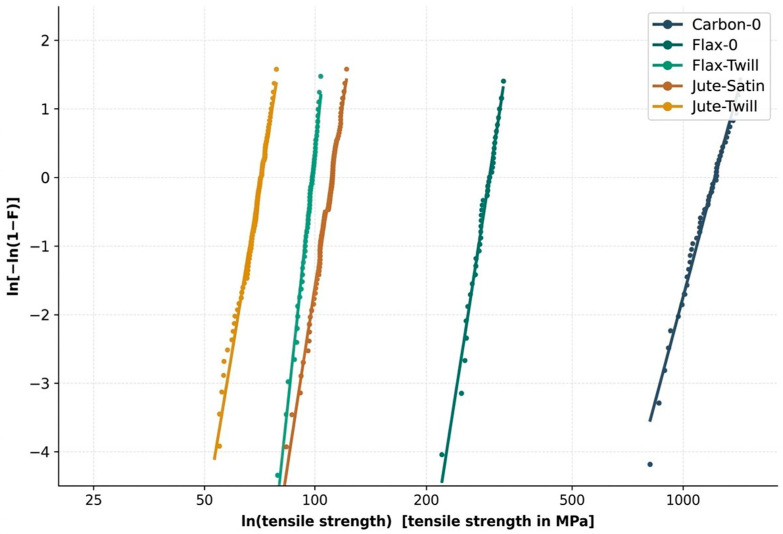
Weibull probability plot for representative tensile-strength groups.

Figure 7 shows the Weibull probability plots for the representative tensile strength groups. The approximately linear trends support the usefulness of Weibull-style lower-tail screening, although the best AIC distribution was not always the Weibull. Weibull analysis has been widely used for tensile-strength reliability in composite materials [16], and fiber-strength statistics can strongly affect composite tensile predictability [17]. The mixed distribution behavior observed here is expected because the composite property distributions can differ according to the architecture, processing route, and failure mode. Representative probability plots for additional material groups are provided in Supplementary Figure S1, complementing the Weibull examples shown in Figure 7 and illustrating all three candidate distribution families considered in this study.

### 4.6. Balanced Lower-Tail Reliability Ranking

**Figure 8:**
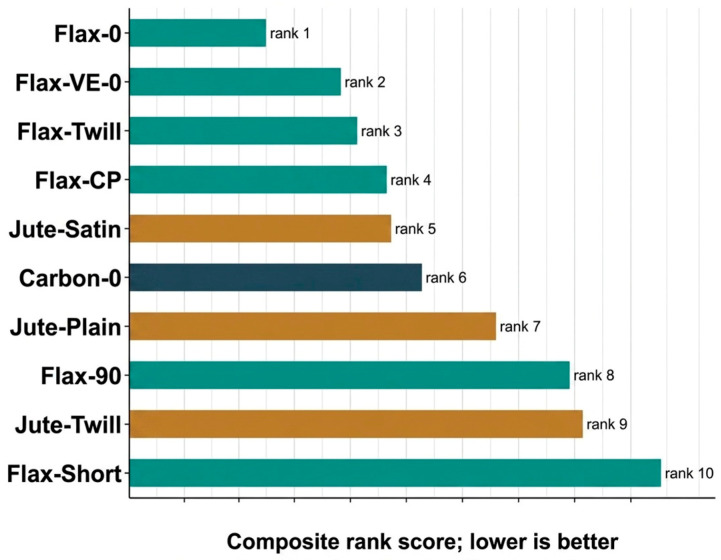
Balanced lower-tail reliability ranking. The score combines the lower-tail ranks and coefficient-of-variation ranks across the three retained properties.

Figure 8 shows the ranking of balanced reliability. Flax-0 ranked first overall, achieving the best combination of a high lower-tail modulus (16,956.66 MPa), high lower-tail tensile strength (268.94 MPa), and a tensile strength CV of only 4.50%, the third lowest among all groups. Flax-VE-0 ranked second because it also delivered strong lower-tail natural fiber performance in both modulus and strength, although its slightly higher CV values (6.92% for strength, 6.98% for modulus) placed it behind Flax-0 in the stability component. Flax twill ranked third owing to its highest lower-tail failure strain (0.0216) and the second lowest tensile strength CV (3.43%), making it the most attractive system where strain capacity and repeatability are simultaneously required. Flax-CP ranked fourth and Jute-Satin fifth; both systems showed consistent distributions, with no single property standing out as a weakness. Carbon-0 ranked sixth in the balanced indices. Although it dominated the lower-tail modulus and strength by a large margin, its lower-tail failure strain (0.0077) was the lowest of all ten systems, and its scatter penalties across failure strain offset the advantages in the strength and stiffness components. This result illustrates that the balanced index is not simply a reordering of the mean properties but a genuinely multidimensional screening tool.

### 4.7. Sensitivity of Reliability Ranking

**Figure 9:**
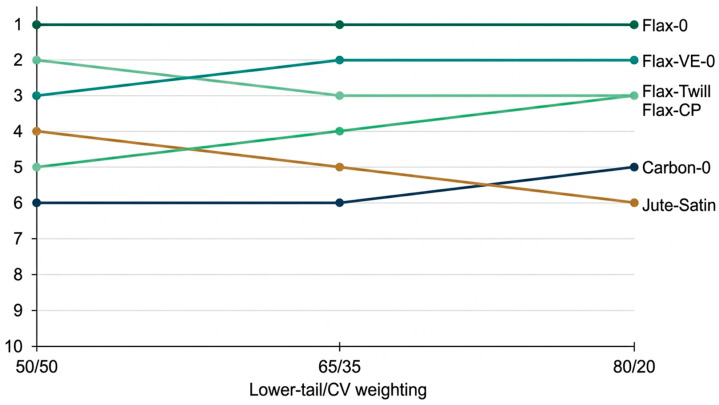
Sensitivity of the balanced reliability ranking to the weighting between lower-tail performance and coefficient of variation stability.

Figure 9 shows the ranking-sensitivity analysis. Flax-0 remained the top-ranked material system under all the tested weighting schemes. Flax-VE-0 and Flax-Twill remain within the top three for the 50/50 and 65/35 cases and remained among the leading natural-fiber systems when the lower-tail contribution increased to 80%. Flax-CP also ranked among the top three under the 80/20 weighting because of its strong lower-tail failure strain and strength balance.

Carbon-0 entered the top five only when the lower-tail performance was weighted most heavily, reflecting its dominant modulus and strength but lower strain-to-failure reliability. The consistent ranking of the leading natural-fiber systems across all three weighting schemes demonstrates that the principal conclusions are robust and not an artefact of a particular weighting choice. In particular, the retention of Flax-0 as the highest-ranked material under each scheme confirms its balanced performance across tensile modulus, tensile strength, and axial failure strain. Nevertheless, the proposed ranking has inherent limitations. As a rank-based index, it does not account for the magnitude of differences between adjacent materials, and its outcome depends on the selected performance criteria and weighting factors. Consequently, the framework should be regarded as a comparative screening tool for preliminary material selection rather than a certified design index. For applications with different performance priorities, the weighting factors in Equation (1) may be adjusted to reflect the specific design requirements.

## 5. Discussion

### 5.1. Mean Performance and Reliability Are Not the Same

The results show that the highest-performing system in absolute terms is not necessarily the most attractive under a balanced reliability criterion. Carbon-0 clearly dominated the tensile modulus and tensile strength. Its empirical lower-tail values were far above those of all the natural-fiber groups. However, its failure strain lower-tail value was lower than those of several flax and jute systems. This reflects the expected trade-off between the high stiffness and strain tolerance. For natural fiber composites, Flax-0 and Flax-VE-0 offered the best lower-tail strength and modulus. These results are consistent with the strong role of fiber alignment in the unidirectional laminates. Flax-Twill and Flax-CP exhibited lower strengths but strong lower-tail failure strains, making them attractive when strain tolerance and progressive tensile response are important. The difference in lower-tail strength between Flax-0 (268.94 MPa) and Flax-VE-0 (241.26 MPa) is modest in absolute terms, but the vinyl ester matrix in Flax-VE-0 showed a slightly higher CV, consistent with published studies suggesting that matrix chemistry can influence the variability of composite properties through differences in fiber–matrix adhesion quality [14]. However, the present dataset does not permit this mechanism to be confirmed directly, and the observed differences may also reflect batch-to-batch variations in fiber properties or minor processing differences. Therefore, this explanation should be regarded as a plausible interpretation rather than a definitive conclusion. Flax-0 with an epoxy matrix is therefore a more reliable choice when both high lower-tail strength and low scatter are required simultaneously. The advantage of fiber alignment in reducing scatter—reflected in the low CVs of Flax-0, Flax-90, and Flax-VE-0 relative to Flax-Short is consistent with the fiber-averaging mechanism reported at the laminate scale, where aligned fibers share the load more uniformly than randomly distributed ones [10]. Carbon-0 exhibited the highest lower-tail tensile modulus and tensile strength among all material groups but ranked lower in the balanced index because of its comparatively low lower-tail failure strain, reflecting the inherently stiff and relatively brittle behavior of carbon/epoxy laminates. Consequently, its excellent stiffness and strength are offset by reduced lower-tail strain performance in the balanced index. This result highlights that the proposed framework rewards balanced reliability across all three retained tensile properties rather than exceptional performance in only one or two properties. Accordingly, the balanced ranking is most appropriately interpreted for comparisons within the natural-fiber composite class, with Carbon-0 included primarily as a synthetic-fiber reference.

### 5.2. Short-Fiber and Twill Systems Show Different Reliability Behavior

Flax-Short exhibited the highest tensile strength CV and high failure strain CV, indicating scatter in both strength and ductility. This is plausible because randomly oriented short fiber mats can be more sensitive to local fiber distribution, fiber length variation, and resin-rich regions. Jute-Twill also exhibited a high failure strain CV, suggesting that its strain-to-failure response is less repeatable than those of Jute-Plain and Jute-Satin. The bootstrap interval for the Jute-Twill failure strain fifth percentile was also broad, indicating the sensitivity of the lower-tail estimate to a small number of low-strain observations. In contrast, flax twill achieved the lowest tensile strength CV among the woven architectures (3.43%) alongside the highest lower-tail failure strain (0.0216), a combination that is unusual among natural fiber systems. This makes Flax-Twill an attractive system for applications where strain capacity and predictable progressive failure are simultaneously required, such as in energy-absorbing panels or compliant structural components. Jute-Satin was the most consistent jute architecture across all three retained properties, combining the highest lower-tail jute strength (98.94 MPa), a strength CV below 5%, and a failure strain CV of 6.83% the lowest among the jute groups. Within the woven jute architectures, the satin weave offers a more favorable reliability profile than plain or twill configurations, consistent with the generally reported tendency of satin weaves to exhibit lower fiber crimp than plain and twill weaves, thereby promoting more uniform load transfer and reducing localized stress concentrations associated with fiber undulation [13]. However, the present dataset does not permit this mechanism to be confirmed directly, and further dedicated multi-batch experimental studies are needed to isolate the influence of weave architecture from other sources of variability, such as fiber quality and processing conditions.

### 5.3. Distribution Fitting Supports Lower-Tail Screening but Is Not Certified Allowable

The tensile strength distribution fits showed that no single distribution was best for all material groups. Normal fits were adequate for several natural-fiber strength groups, whereas Weibull fits were selected for Carbon-0, Flax-90, Flax-VE-0, and Jute-Twill. For the other retained properties, the AIC-selected distributions differed by material group; for example, lognormal fits were frequently competitive for the modulus. The predominance of lognormal fits for the tensile modulus across nine of ten groups is consistent with the log-additive nature of the modulus scatter in composite systems, where variability arises from the multiplicative contributions of fiber volume fraction, fiber alignment, and local geometry [15]. In contrast, the mix of normal, lognormal, and Weibull fits for the failure strain reflects the greater complexity of the failure process, which can involve matrix microcracking, fiber pull-out, and interfacial debonding in proportions that vary with architecture [16]. The practical implication is that modulus lower-tail estimates can be made with reasonable confidence using lognormal fits, whereas failure strain lower-tail estimates require individual distribution screening for each material group. This mixed behavior supports the use of distribution screening rather than assuming a universal form. The identification of different best-fit distributions across the material groups reflects genuine differences in the statistical characteristics of the respective material–property combinations rather than a limitation of the analytical approach. By selecting the most appropriate distribution for each group using the AIC criterion, the analysis provides more representative lower-tail estimates than would be obtained by imposing a single distribution model across all materials. Consequently, the resulting fifth percentile values remain directly comparable as consistent measures of lower-tail performance across the investigated composite systems.

The fifth percentile values reported in this study should be interpreted carefully. They are useful lower-tail descriptors for comparing groups in research datasets. These are not formal design allowable. Certified A-basis or B-basis allowable require stricter sampling plans, batch tracking, statistical procedures, and acceptance criteria [11,16]. The CV values observed in this dataset are broadly consistent with, and in several cases lower than those reported in the literature. For flax fiber laminates, published tensile strength CV values typically range from approximately 4% to 16%, depending on the architecture, processing route, and fiber supply [6,10]. The present Flax-0 tensile-strength CV of 4.50% and Flax-Twill CV of 3.43% are near the lower boundary of this published range, indicating that the Torres et al. dataset captures well-controlled test conditions. For jute systems, tensile strength CV values below 5% as observed for Jute-Satin (4.83%) and Jute-Plain (4.92%) in this study are consistent with the better-performing woven jute laminates reported in the broader literature, where scatter tends to be higher in short-fiber or randomly oriented jute architectures [1,2]. The Flax-Short CV of 13.42% also aligns with the expectations for non-woven short-fiber systems, which are reported to exhibit substantially higher scatter than aligned or woven architectures owing to random fiber placement and resin-rich zone formation [6]. These comparisons support the interpretation that the present dataset is representative of the natural fiber composite class rather than atypically uniform and that the reliability rankings reported here reflect genuine material behavior.

### 5.4. Relationship with the Original Dataset and Previous Work

Torres et al. established the statistical behavior of natural fiber composite laminates and showed that well-processed long natural-fiber laminates can exhibit variability comparable to carbon fiber systems when tested under controlled conditions [10]. That study reported group-level means and standard deviations and concluded that architecture and fiber type were the dominant sources of property variations. The present work extends the dataset-level understanding in three specific directions: first, by computing empirical lower-tail percentiles and bootstrap confidence intervals that provide a reliability-focused perspective absent from the original study; second, by performing distribution screening via AIC to determine whether normal, lognormal, or Weibull forms best describe each group-property combination; and third, by synthesizing the multi-property behavior into a single balanced reliability ranking with documented sensitivity. The analysis was not a predictive machine learning study. Its contribution is in material screening and reliability interpretation, offering a complementary perspective to mean property comparisons and prediction-oriented modeling approaches.

### 5.5. Scope of Interpretation and Robustness

The dataset was generated under a consistent experimental program, providing a robust basis for comparative analysis because all material systems were evaluated under common testing and processing conditions. Accordingly, the present study should be interpreted as a benchmark assessment of lower-tail reliability rather than as a certified design allowable study. The reported fifth percentile values are lower-tail statistical descriptors and should not be interpreted as formal A- or B-basis allowables, which require dedicated qualification protocols and additional statistical requirements. To improve the robustness of the analysis, bootstrap confidence intervals, AIC-based distribution screening, and sensitivity analyses of the balanced reliability ranking were performed.

The sensitivity analysis demonstrated that the principal natural-fiber rankings were robust across all weighting schemes, with Flax-0 consistently ranked first and Flax-VE-0 and Flax-Twill remaining among the leading systems. The specimen counts per group (n = 37–87) are appropriate for the comparative statistical screening performed in this study. For context, ASTM D3039 specifies a minimum of five specimens for basic tensile property evaluation, whereas statistical B-basis allowable development typically requires 30 or more specimens per batch. Accordingly, the present dataset is suitable for lower-tail benchmarking and distribution screening, although larger independent multi-batch datasets would be required for formal design allowable development. Another limitation is that the present analysis considers only dry-condition tensile properties. Natural-fiber composites are known to be sensitive to environmental effects such as moisture absorption, which can reduce tensile strength and stiffness while increasing property scatter through fiber swelling and fiber–matrix debonding [18]. Consequently, the lower-tail rankings reported here should be regarded as dry-condition benchmarks. Future work should extend the proposed framework to additional natural-fiber systems, multi-batch qualification datasets, environmentally conditioned composites, and hybrid natural/synthetic-fiber laminates to evaluate lower-tail reliability under a broader range of material systems and service conditions.

## 6. Conclusions

This study presents a statistical variability and lower-tail reliability analysis of 590 tensile specimens from flax, jute, and carbon fiber composite laminates. The analysis focused on the recalculated tensile modulus, tensile strength, and axial failure strain. The main conclusions are as follows:

1. Carbon-0 exhibited the highest lower-tail tensile modulus (104.95 GPa) and tensile strength (989.64 MPa), confirming the superior performance of the unidirectional carbon/epoxy reference system. Among the natural-fiber composites, Flax-0 (16.96 GPa; 268.94 MPa) and Flax-VE-0 (13.60 GPa; 241.26 MPa) showed the highest lower-tail modulus and strength, whereas Flax-Twill (P_5_ = 0.0216) and Flax-CP (P_5_ = 0.0199) demonstrated the best lower-tail failure-strain performance.
2. Five natural-fiber systems (Flax-90, Flax-Twill, Flax-0, Jute-Satin, and Jute-Plain) achieved tensile-strength coefficients of variation below 5%, demonstrating that well-designed natural-fiber laminates can achieve mechanical consistency comparable to industrial carbon-fiber composites. In contrast, Flax-Short exhibited the highest strength variability (CV = 13.42%), reflecting the greater scatter associated with randomly oriented short-fiber architectures.
3. No single statistical distribution adequately described all material–property combinations. Instead, the optimal distribution depended on both the material system and tensile property, demonstrating that independent AIC-based distribution screening is necessary for reliable lower-tail performance assessment rather than assuming a universal distribution.
4. The balanced lower-tail ranking (65% empirical fifth percentile and 35% CV) consistently identified Flax-0, Flax-VE-0, Flax-Twill, Flax-CP, and Jute-Satin as the most promising natural-fiber systems. The ranking remained stable across all weighting schemes evaluated, demonstrating the robustness of the proposed framework for lower-tail reliability assessment and preliminary material selection beyond conventional mean-property comparisons.

This study provides a reproducible, reliability-focused analysis of an open natural-fiber composite tensile dataset. This is distinct from prediction-oriented machine learning work and can support material selection where lower-tail behavior is important.

## Figures and Tables

**Table 1:** Tensile strength lower-tail descriptors and distribution screening.

Material Group	n	Best Distribution	Mean Strength (MPa)	CV (%)	Empirical Fifth Percentile (MPa)	Model Fifth Percentile (MPa)
Carbon-0	46	Weibull	1176.11	8.54	989.64	979.09
Flax-0	40	Normal	288.58	4.50	268.94	267.19
Flax-90	37	Weibull	31.17	2.41	29.58	29.67
Flax-CP	68	Normal	152.70	5.44	141.88	139.05
Flax-Short	41	Lognormal	46.76	13.42	37.13	37.11
Flax-Twill	54	Normal	95.81	3.43	88.79	90.40
Flax-VE-0	45	Weibull	274.20	6.92	241.26	237.36
Jute-Plain	86	Normal	71.81	4.92	66.28	66.00
Jute-Satin	87	Normal	107.87	4.83	98.94	99.30
Jute-Twill	86	Weibull	68.22	5.34	61.75	61.26

n = number of specimens per group. All groups without an explicit resin designation use an epoxy thermoset matrix. Flax-VE-0 is the only vinyl ester matrix group.

**Table 2:** Distribution screening summary for the tensile modulus and axial failure strain.

Material Group	Modulus Best Distribution	Empirical Fifth Percentile Tensile Modulus (GPa)	Failure-Strain Best Distribution	Empirical Failure-Strain Fifth Percentile
Carbon-0	Lognormal	104.95	Weibull	0.0077
Flax-0	Normal	16.96	Normal	0.0172
Flax-90	Lognormal	3.77	Weibull	0.0074
Flax-CP	Lognormal	6.88	Weibull	0.0199
Flax-Short	Lognormal	3.58	Lognormal	0.0084
Flax-Twill	Lognormal	5.68	Normal	0.0216
Flax-VE-0	Lognormal	13.60	Weibull	0.0183
Jute-Plain	Lognormal	5.62	Weibull	0.0114
Jute-Satin	Weibull	7.33	Lognormal	0.0150
Jute-Twill	Lognormal	5.62	Weibull	0.0098

## Data Availability

The original contributions presented in this study are included in the article/Supplementary Material. Further inquiries can be directed to the corresponding authors.

## Supplementary Materials

The following supporting information can be downloaded at: https://www.mdpi.com/article/10.3390/polym18141746/s1, Supplementary Figure S1: Representative normal and lognormal probability plots for tensile-strength data.
