# Supplementary material for: Statistical Variability and Lower-Tail Performance Assessment of Tensile Properties in Flax, Jute, and Carbon Fiber Composite Laminates
Source: Polymers (Basel). 2026 Jul 16;18(14):1746. doi: 10.3390/polym18141746 (PMC13416983; doi:10.3390/polym18141746)
Supplement: Supplementary file 1 [file polymers-18-01746-s001.zip › polymers-4413615-supplementary.pdf]

Supplementary Figure S1

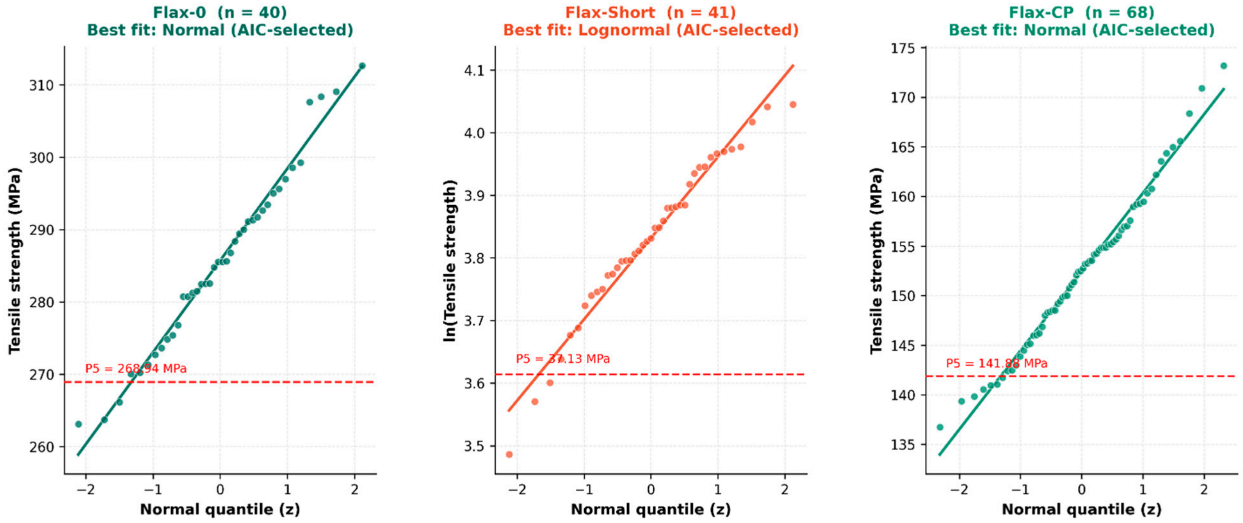

Figure S1. Representative normal and lognormal probability plots for tensile-strength data. Flax-0 and Flax-CP are best described by the normal distribution, whereas Flax-Short is best described by the lognormal distribution according to the AIC-based distribution screening. The dashed horizontal line indicates the empirical 5th percentile (P_5). Together with the Weibull probability plots presented in Figure 7, these examples illustrate all three candidate distribution families evaluated in this study.
